# Fatal Road Traffic Accidents and Associated Factors in North Shewa Zone, Central Ethiopia: A Cross-Sectional Study

**DOI:** 10.4314/ejhs.v33i6.7

**Published:** 2023-11

**Authors:** Tilahun Deresse, Akine Eshete, Minyahil Hailu, Megbar Dessalegn

**Affiliations:** 1 Department of Surgery, School of Medicine, Debre Berhan University, Debre Berhan, Ethiopia; 2 Department of Public Health, Debre Berhan University, Debre Berhan, Ethiopia; 3 Department of Surgery, School of Medicine, Debre Markos University, Debre Markos, Ethiopia

**Keywords:** Magnitude, road traffic accident, North Shewa zone, Ethiopia

## Abstract

**Background:**

Road traffic accidents (RTAs) are among the top three global causes of death among people aged 15 to 44 years. More importantly, it is the main cause of death and permanent disability among young people aged 15 to 29 years. This study aimed to assess the magnitude of fatal traffic accidents and the factors associated with them in the North Shewa Zone, Central Ethiopia, from 2013 to 2018.

**Method:**

An institution-based cross-sectional study was conducted in all registered RTAs from July 2013 to June 2018 that had full documentation. The data extraction tool was developed based on the daily RTA registration book format that was utilized. Data was entered into Epi-data version 3.1 and then exported to SPSS version 21 for analysis. Logistic regression analysis was used to assess the relationship between the factors and the fatality of RTA. P-values less than 0.05 were reported as statistically significant.

**Results:**

Among 846 RTAs studied, 351 (41.5%) were found fatal, while 495 (58.5%) caused non-fatal injuries. Failure to give priority to pedestrians was 2.8 times (AOR = 2.8, 95% CI: 1.3, 5.9) more likely to cause fatal RTAs than drivers who failed to maintain distance between vehicles. Pedestrians were 2.7 times (AOR = 2.7, 95% CI: 1.1, 6.7) more likely to die in RTAs than drivers.

**Conclusion:**

The fatality of RTA was high. Failure to give priority to pedestrians and being a pedestrian were strong predictors of death. The North Shewa Zone Traffic Police Department and police officers should focus on enforcing traffic safety laws.

## Introduction

Vehicles are the ultimate human innovation that makes life easy. However, incongruous usage has resulted in the loss of irreplaceable lives in road traffic accidents. A road traffic accident is defined as an incident that involves at least one moving vehicle, takes place on a public road or street, and results in one or more fatalities or serious injuries. Collisions between cars, between vehicles and pedestrians, between vehicles and animals, or between vehicles and fixed obstacles constitute a road traffic accident. Road traffic injury, which is physical damage to a person as a result of a road traffic crash, is the major cause of traffic fatalities (any person killed immediately or dying within 30 days as a result of a road traffic injury) (1).

One of the top causes of fatalities and permanent disabilities worldwide is traffic-related injury. Road traffic accidents are listed as one of the main causes of death for all age groups and are reported to be the leading cause of death among younger adults aged 15 to 29 years. Compared to HIV/AIDS, TB, and diarrheal diseases combined, road traffic accidents claim more lives. In 1990, 2010, and 2013, respectively, the Institute for Health Metrics and Evaluation (IHME) projected approximately 907,900, 1.3 million, and 1.4 million deaths from traffic injuries (2).

High-income nations have the lowest regional death rate (8.7 per 100,000 people involved in road traffic accidents (3), while middle-income and low-income countries suffer from high death rates (20.1 per 100,000 and 18.3 per 100,000, respectively) (3). There are discrepancies, not only where people die, but also who dies. Although there are large differences between European countries, the probability of death from traffic accidents is lowest in Europe and the greatest in Africa. In Africa, 38% of deaths are among pedestrians, while in the Western Pacific region, 36% of deaths are among motorcyclists, shadowing the preferred mobility options in these two regions (4). Reasons for the high burden of road traffic-related deaths and injuries in developing countries were related to factors like rising vehicle ownership, lax enforcement of traffic laws, inadequate public health infrastructure, and limited access to healthcare compared to developed nations. In addition to the humanitarian impact of the issue, these nations lose between $65 billion and $100 billion annually as a result of traffic-related deaths and injuries. In addition to losing wages, families are also required to pay for the costs associated with caring for injured family members. Economic losses of one to two percent of the gross domestic product are predicted to occur in low- and middle-income nations (1, 4, 5).

Beyond the immense suffering they cause, traffic accidents put a family in poverty because the survivors and their families find it difficult to deal with the long-term effects of the events. The cost of medical care and rehabilitation, funeral costs, and the loss of the family breadwinner are among these factors. The national health system might also be severely strained as a result of road traffic injuries (5).

As the primary mode of transportation, roads in Ethiopia have seen a high incidence of traffic accidents. The number of fatal traffic accidents per 10,000 motor vehicles from 2002 to 2005 was between 129 and 145 (4). Furthermore, Ethiopia is suffering from these high traffic accident records despite having a low road network density and vehicle ownership (6).

Along with having poorly built roadways, which increase the risk of accidents involving cyclists and pedestrians, the current roads are not regularly inspected to maintain them on time. The national speed limit policy is poorly implemented, and it is carried out manually by people, which combined with other factors, contributed to a startling increase in the number of fatal road traffic accidents between the years 2008 and 2016 (4). As a result, road traffic deaths and injuries have been among the primary challenges to the nation's public health and development, and they will continue to negatively impact community well-being and the nation's economy unless serious action is taken to address the issue (6).

There were few studies in North Shewa Zone, Amhara National Regional State (ANRS), related to road traffic accidents (7). However, these studies focus on general outcome severity. Determining the magnitude and primary factors that contribute to fatality from traffic accidents, using pooled data in the North Shewa Zone, was the purpose of this study.

## Materials and Methods

The study was done from March, 1 – April 30, 2019, in Debre Berhan, Amhara region, Ethiopia. North Shewa is one of the administrative zones found in the Amhara regional state, 130 km northeast of Addis Ababa, the capital city of Ethiopia, and 695 km away from the capital city of Amhara region, Bahir Dar. Generally, the North Shewa zone has 27 woredas (districts) and 442 kebeles. According to the information obtained from the zonal vital events registration office, in 2017/18, the total population of the zone was 2,263,097. Debre Berhan serves as the zone's capital city and is connected to Ethiopia's northern regions via a road that runs through the town from Addis Ababa. This area has one of the highest rates of road traffic accidents due to the combination of the area's challenging geographic location and being one of the busiest roads in the nation.

This study employed an institution-based cross-sectional design by including all road traffic accidents reported to the North Shewa Zone Police Department between July 2013 and June 2018. It used all the necessary data in the registry. Thus, 890 documented registries of RTAs were reported in the period. Among these, 44 had incomplete registries and were excluded from the analysis. Thus, the study was conducted on 846 RTA registries.

The data extraction tool was developed based on the daily RTA registration book format, and data was collected by three nurses who were trained by the principal investigator. Data collection was supervised, and the quality of data was checked every day.

Extracted data were entered into Epi-data version 3.1, exported to, cleaned, and analyzed using SPSS version 21. The assumptions of the chi-square test were duly verified and deemed to have been met before its execution, while the model fitness test was examined before logistic regression analysis. Binary logistic regression was used to assess the associations between the fatality of RTAs and related socio-demographic, driver-related, vehicle-related, and environmental factors. Factors with p-values less than 0.25 in bivariate analysis were included in the multivariable binary regression. Variables with a p-value < 0.05 were reported as statistically significant. In this study, a road traffic accident is defined as physical damage to a person as a result of a road traffic crash, and a fatal road traffic accident happens when any person is killed immediately or dies within 24 hours as a result of a road traffic crash injury.

**Ethical approval and consent:** Ethical clearance was obtained from the Ethical Review Board of Debre Berhan University, Asrat Waldeyes Health Science Campus (IRB-137). No personal identifiers were included in the records during data extraction. Therefore, the study did not inflict any harm on individuals. All information used from the registries was kept confidential, and all activities were performed according to the relevant guidelines and regulations.

## Results

**Socio-demographic characteristics of drivers and victims of road traffic accidents**: In this study, of the total of 890 road traffic accidents (RTAs) that were registered between July 2013 and June 2018 in the North Shewa Zone Traffic Police Department in Central Ethiopia, 846 (95.05%) complete records were analyzed. Drivers between the ages of 31 and 50 were responsible for the majority (44.2%) of collisions, while drivers between the ages of 19 and 30 were responsible for 38.9% of collisions. Only 19% and 66.2% of the drivers had completed elementary and high school, respectively. Drivers constituted nearly half of the victims in fatal RTAs, 173 (49%), whereas the rest were passengers (46%), and pedestrians (16%). Road traffic accidents from private vehicles constituted the majority (95.2%), while the remaining were from government-owned vehicles (Table 1).

**Table 1:** Socio-demographic characteristics of drivers involved in road traffic accidents, North Shewa zone, Amhara regional state, Central Ethiopia, from July 2013 to June 2018 (N=846)

Variables and Categories	Frequency (%)
**Driver Age**	
≤18	53 (6.3)
19-30	329 (38.9)
31-50	374 (44.2)
50+	90 (10.6)
**Educational status**	
Grade 1-8	161 (19)
Grade 9 -12	560 (66.2)
Diploma	115 (13.6)
Degree and above	10 (1.2)
**Marital status**	
Single	439 (51.9)
Married	383 (45.3)
Divorced	19 (2.2)
Widowed	5 (0.6)
**Driving Experience**	
< 1 year	200 (23.6)
1-3 year	212 (25.1)
4-5 years	218 (25.5)
6- 10 years	103 (12.2)
10+ years	113 (13.4)
**Vehicle ownership**	
Private	805 (95.2)
Government	41 (4.2)
**Type of victims**	
Passengers	446 (52.7)
Pedestrian	27 (3.2)

Drivers only	373 (44.1)

**Drivers' behavior and related factors in Road traffic accidents**: Among all accidents, 307 (36.7%) occurred on the straight road and nearly 22% occurred in cloudy and rainy seasons. Twenty-three percent of the accidents were attributed to exceeding the posted speed limit, while about 20% were due to pedestrians not being given priority. The remaining causes of road traffic accidents in the area included driving on the wrong side of the road, failing to wear a seatbelt, driving for several hours without a break (fatigue), not giving priority to other vehicles, and failing to keep distance between vehicles (Table 2).

**Table 2:** Road traffic accidents according to road conditions, weather conditions, and driver behaviors in North Shewa Zone, Amhara Regional State, Central Ethiopia, from July 2013 to June 2018

Variables and Categories	Frequency
**Road condition (846)**	
Strait	307 (36.3)
Curved	276 (32.6)
Little curved	138 (16.3)
Downward	93 (11.0)
Upward	32 (3.8)
**Road type (846)**	
Asphalt	698 (82.5)
Gravel road	148 (17.5)
**Weather condition (846)**	
Dry	575 (68.0)
Cloudy	151 (17.8)
Rainy	84 (9.9)
Cold	13 (1.5)
Windy	13 (1.5)
Hotter	10 (1.2)
**Drivers related causes (846)**	
Failure to give priority to	164 (19.4)
pedestrian	
Failure to give priority to other	51 (6.0)
Over speed	195 (23.0)
Driving for several hours	52 (6.1)
Negligence	85 (10.0)
Driving on the wrong side of the	156 (18.4)
Not using a seat belt	93 (11.0)
Failure to maintain distance b/n	50 (5.9)
vehicles	
**Driving license (846)**	
No driving license	75 (8.9)
1st level	6 (0.7)
2nd level	11 (1.3)
3^rd^ level	87 (10.3)
4th level	63 (7.4)
5th level	48 (5.7)
Taxi 1	43 (5.1)
Public 1	260 (30.7)
public 2	69 (8.2)
solid 1	73 (8.6)
Solid 2	72 (7.5)
Solid 3	39 (4.6)

**Figure 1:**
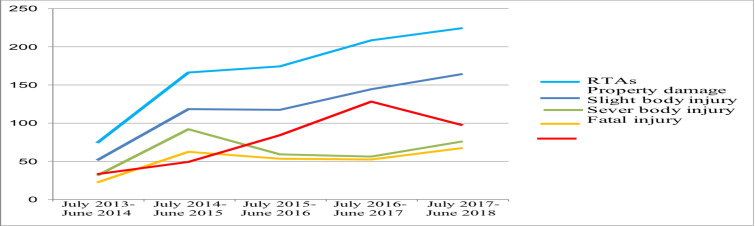
Trends of RTA in North Shewa Zone, Amhara Regional State, Central Ethiopia, July 2013 – June 2018

**The Magnitude and pattern of road traffic accidents**: Out of the 846 RTAs analyzed, 351 accidents (41.5%) resulted in fatalities, while non-fatal accidents occurred in 495 of the cases. Over a five-year study period, the number of RTAs increased from 74 to 224. Between July 2017 and June 2018, there were 224 RTAs, which account for 26.5% of all RTAs encountered. A parallel increment in mortality was seen over the first four years attaining its peak at the fourth year with 128 deaths, before subsequent reduction during the period of July 2017 to June 2018, to 97 deaths (Figure 1).

**Factors associated with fatal road traffic accidents**: Variables exhibited a significant association with the fatality rate of road traffic accidents in the multivariate analysis. Younger drivers (aged 18 and below) were at increased risk of fatal road traffic accidents. They more than doubled the risk of mortality compared to older drivers (AOR: 2.5 (1.22, 5.24)). Pedestrians were 2.7 times (AOR: 2.7, 95% CI: 1.10, 6.74) more likely to die in a road traffic accident than drivers (Table 3). The likelihood of being fatal was significantly predicted by failure to give priority to pedestrians. This increased the likelihood by 2.8 times (AOR: 2.8, 95% CI: 1.3, 5.9). Road traffic accidents that happened in asphalt roads were found less likely to be fatal compared with non-asphalt (Gravel roads) roads (AOR: 0.6, 95% CI: 0.41–0.91).

**Table 3:** Factors associated with fatality of road traffic accidents from multivariate analysis, in North Shewa Zone, Amhara Regional State, Central Ethiopia, from July 2013 to June 2018

Variables	Category	Fatal	Non-	COR (95% CI)	AOR (95% CI)
Drivers age	≤ 18	31	22	3.1(1.54,6.32)*	2.5(1.22, 5.24)
	19 – 30	146	183	1.7 (1.08,2.90)*	0.9(0.26, 2.91)
	31-50	146	228	1.4(0.87, 2.32)	0.8(0.23,2.64)
	50-65	28	62	1	1
Driving license	No license	44	31	3.8(1.62,9.05)*	1.4(0.28, 7.02)
	First level	5	1	13.5(1.40,130.19)*	9.1(0.8,102.39)
	Second level	5	6	2.2(0.56,9.04)	0.9(0.26, 2.91)
	Third level	37	50	1.9(0.86,4.63)*	0.8(0.22,2.64)
	Taxi 1	30	13	6.2(2.35,16.51)	2.5(0.8,7.55)
	Public 1	103	157	1.8(0.82,3.81)*	0.7(0.31,1.55)
	Solid 1	30	43	1.9(0.79,4.46)*	0.7(0.29,1.50)
	Solid 2	29	43	1.8(0.77,4.32)*	1.8(0.88,3.63)
	Solid 3	10	27	1	1
Type of victim	Passenger	162	284	0.66(0.49,0.87)*	1.1(0.58,2.04)
	Pedestrians	16	11	1.7(0.76,3.72)	2.7(1.10,
	Driver	173	200	1	1
Driver-related	Failure to give priority to pedestrian	108	56	2.7(1.39,5.09)*	2.8(1.36,5.93)**
causes of RTA	Failure to give priority to other vehicles	17	34	0.7(0.31,1.55)	0.9(0.26, 2.91)
	Speed	86	109	1.1(0.58,2.04)	0.8(0.23,2.64)
	Fatigue/driving for several hours	17	35	0.7(0.29,1.50)	0.8(0.24, 3.03)
	Negligence	48	37	1.8(0.88,3.63)*	1.4(0.28, 7.02)
	Driving on the wrong side of the	39	117	0.5(0.24,0.90)*	0.5(0.25, 1.07)
	Failure to maintain distance	21	29	1	1
	between vehicles				
Road type	Asphalt	271	427	0.5(0.38, 0.77)*	0.6(0.41,0.91)**
	
	Gravel Road	80	68	1	1

## Discussion

The purpose of this study was to assess the magnitude of fatal traffic accidents and the factors associated with them in the North Shewa zone, Central Ethiopia, from 2013 - 2018. Road traffic accidents have emerged as a significant economic and public health concern on a global scale, and they are worsening in emerging nations like Ethiopia. This study demonstrated a significant (N = 846) and upwardly trending number of accidents that occurred in the North Shewa Zone over five years. The fatality rate of RTA (41.5%) identified in this study was greater than the rates in Burayu, Oromia, Central Ethiopia (29.8%), Rwanda (18.5%), Vietnam (21%), and France (36%) (8-11). This difference might be due to the recent robust increase in traffic flow due to industrialization and disproportionately high population growth of the zone (mainly due to the inflow of people from all corners of the country).

Two hundred and twenty-four accidents happened from July 2017 to June 2018, which is three times higher compared with the number of accidents that happened from July 2013 to June 2014, 74 accidents. This finding of increasing trend in number of accidents is consistent with other studies in Ethiopia (7,10,12-16). Similarly, Singh et al. indicated a steady rise in the frequency of road traffic accidents in India (17). As towns grow and numerous industrial parks are built (18), there could be an exponential rise in the number of vehicles and traffic flow entering the area, which could be one of the main causes of the RTAs' growing tendency (5,17,19). Additionally, the area's stunning and rugged environment, lax enforcement of traffic safety laws, and ineffective car technical checks could all be making the issue worse (5,18).

This study demonstrated that drivers (49%) and passengers (46%) make up the majority of fatal RTA casualties, as opposed to pedestrians (5%) as in studies carried out in Northwest Ethiopia (7) and other low and middle-income nations (1, 2, 4, 5, 20). This could be attributed to the frequent use of buses for transportation, which usually board twice as many passengers as possible due to lax enforcement of traffic safety laws. Although the types of vehicles were not fully documented in the registries, the observation that the majority of drivers, 494 (58.4%) involved in the RTAs, have licenses used to operate buses and minibuses (second level to bus 2 licenses), might provide additional evidence for the aforementioned explanation about the significant number of passengers' death. The rise in the number of lorries in the region, which are typically driven on incredibly rough roads in the rural portions of the zone to transport raw materials to industry might explain why more drivers are dying on the road (18).

This study showed that failing to give priority to pedestrians, road type where the accident occurred, and involvement of pedestrians in the accident were significant predictors of road traffic fatalities in the North Shewa Zone, which is consistent with other similar studies carried out in Low-Middle-Income-Countries (LMICs) (4, 5, 6, 7, 10-18, 21, 22).

This study found no evidence of a statistically significant association between drivers' ages and fatal or non-fatal RTAs, in contrast to earlier studies of a similar nature carried out in other regions of Ethiopia that found a statistically significant association (the younger the age (18-30), the higher the occurrence) (6, 7, 10-18, 21, 22). This might be attributed to the area's predominance of lorries and buses, which require more training and experience to operate legally than automobiles and light cars, so the majority of drivers involved in RTAs in this area are beyond the age of 30, 464 (54.8%).

This study has revealed that most of the RTAs were caused by drivers' errors, like failing to give priority to pedestrians (19.4%), driving on the wrong side of the road (18.4%), and not using a seat belt (11%), which is in line with a study done in Burayu, Central Ethiopia (12).

In conclusion, over the specified five fiscal years, the trajectory of road traffic accidents in the North Shewa Zone has shown a gradual rise. Younger people and pedestrians constituted the majority of those involved in traffic accidents. The majority of collisions were the result of errors made by drivers. The Traffic Police Department of the zone should enforce regulations concerning the issuance of licenses, the prohibition of violating safety regulations while driving, and the use of safety belts. Community leaders, non-governmental and governmental institutions, religious institutions, and schools in collaboration with the traffic office should engage in activities that create public awareness and massive behavioral modification about road traffic accidents.
